# Low levels of WRAP53 predict decreased efficacy of radiotherapy and are prognostic for local recurrence and death from breast cancer: a long‐term follow‐up of the SweBCG91RT randomized trial

**DOI:** 10.1002/1878-0261.13426

**Published:** 2023-04-19

**Authors:** Moa Egelberg, Tommaso De Marchi, Gyula Pekar, Lena Tran, Pär‐Ola Bendahl, Axel Stenmark Tullberg, Erik Holmberg, Per Karlsson, Marianne Farnebo, Fredrika Killander, Emma Nimeús

**Affiliations:** ^1^ Division of Surgery, Department of Clinical Sciences Lund, Faculty of Medicine Lund University Sweden; ^2^ Division of Oncology and Pathology, Department of Clinical Sciences Lund, Faculty of Medicine Lund University Sweden; ^3^ Department of Radiology Kristianstad Hospital Sweden; ^4^ Department of Oncology, Institute of Clinical Sciences, Sahlgrenska Academy, Sahlgrenska University Hospital University of Gothenburg Sweden; ^5^ Department of Bioscience and Nutrition & Department of Cell and Molecular Biology Karolinska Institutet Stockholm Sweden; ^6^ Division of Oncology and Pathology, Department of Clinical Sciences Lund, Faculty of Medicine Skåne University Hospital Lund Sweden; ^7^ Division of Surgery, Department of Clinical Sciences Lund, Faculty of Medicine Skåne University Hospital Lund Sweden

**Keywords:** breast cancer, DNA repair, prediction, prognosis, radiotherapy, WRAP53

## Abstract

Downregulation of the DNA repair protein WD40‐encoding RNA antisense to p53 (WRAP53) has been associated with radiotherapy resistance and reduced cancer survival. The aim of this study was to evaluate WRAP53 protein and RNA levels as prognostic and predictive markers in the SweBCG91RT trial, in which breast cancer patients were randomized for postoperative radiotherapy. Using tissue microarray and microarray‐based gene expression, 965 and 759 tumors were assessed for WRAP53 protein and RNA levels, respectively. Correlation with local recurrence and breast cancer‐related death was assessed for prognosis, and the interaction between WRAP53 and radiotherapy in relation to local recurrence was assessed for radioresistance prediction. Tumors with low WRAP53 protein levels had a higher subhazard ratio (SHR) for local recurrence [1.76 (95% CI 1.10–2.79)] and breast cancer‐related death [1.55 (1.02–2.38)]. Low *WRAP53* RNA levels were associated with almost a three‐fold decreased effect of radiotherapy in relation to ipsilateral breast tumor recurrence [IBTR; SHR 0.87 (95% CI 0.44–1.72)] compared with high RNA levels [0.33 (0.19–0.55)], with a significant interaction (*P* = 0.024). In conclusion, low WRAP53 protein is prognostic for local recurrence and breast cancer‐related death. Low *WRAP53* RNA is a potential marker for radioresistance.

## Graphical abstract

We have studied the levels of WRAP53, a DNA repair protein, in 965 breast tumors and correlated this with the prognosis and effect of radiotherapy treatment. Tumors with low levels of WRAP53 had a higher risk for locally recurrent cancer and death from breast cancer. Low levels of *WRAP53* RNA indicated less effect of radiotherapy.

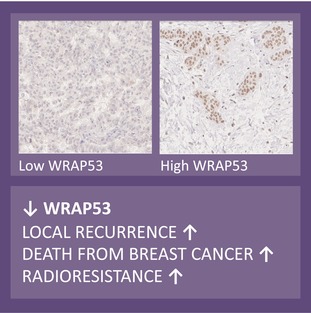

**Abbreviations**
BCD: breast cancer death
CI: confidence interval
ER: estrogen receptor
GSEA: gene set enrichment analysis
HER2: human epidermal growth factor receptor 2
IBTR: ipsilateral breast tumor recurrence
NES: normalized enrichment score
PgR: progesterone receptor
Ref: reference
RT: radiotherapy
SD: standard deviation
SHR: subhazard ratio
TMA: tissue microarray
WRAP53: WD40‐encoding RNA antisense to p53

## 1 Introduction

Breast cancer is the most common malignancy in women worldwide [1] and treatment recommendations are becoming more personalized. Radiotherapy is given after breast‐conserving surgery and roughly halves the incidence of recurrent cancer [2]. Biomarkers predictive of radioresistance is needed in order to modify treatment strategies for patients that develop locoregional recurrence despite radiotherapy.

A potential biomarker of radiotherapy resistance is the protein WRAP53 (alias TCAB1, WDR79, and WRAP53β). WRAP53 is a scaffold protein engaged in the repair of double‐strand DNA breaks [3, 4, 5, 6], the formation and function of Cajal bodies [7], as well as telomerase activity [8]. Previous reports have located WRAP53 in both the nucleus and the cytoplasm of tumor cells [7, 9]. The *WRAP53* gene also gives rise to another transcript, *WRAP53α*, which is antisense to p53 mRNA that increases the levels of p53 in response to DNA damage [10]. Increased levels of nuclear WRAP53 protein have been detected in head and neck squamous cell carcinoma [11], ovarian epithelial cancer [12], rectal adenocarcinoma [13], esophageal squamous cell carcinoma [14], as well as in breast cancer [15]. It has been hypothesized that this increased expression is a natural response to the accumulating amounts of DNA damage known to occur in cancer cells rather than WRAP53 having oncogenic properties. Moreover, it appears like loss of WRAP53 in later stages of tumor evolution leads to the inactivation of the DNA damage response, survival of the genetically unstable tumor cell, and cancer progression [16].

Low levels, in contrast to high levels, of nuclear WRAP53 protein have been associated with impaired survival in both breast [15] and ovarian cancer [12]. However, the association between WRAP53 and locally recurrent breast cancer has not been studied. Moreover, in head and neck cancer low levels of nuclear WRAP53 have been shown to be more common among patients classified as nonresponders to radiotherapy than among responders [11]. While the lack of nuclear WRAP53 protein could be an important prognostic and treatment‐predictive biomarker, its relation to radiotherapy resistance in breast cancer has yet to be investigated.

The aim of the current study was to evaluate the prognostic and predictive roles of WRAP53 protein levels and *WRAP53* RNA expression in SweBCG91RT. In this trial, early‐stage breast cancer patients were randomized to postoperative radiotherapy or not, after breast‐conserving surgery. As few patients have received any other adjuvant therapy it allows for analysis of prognostic and predictive value from radiotherapy alone [17, 18, 19]. The endpoints for prognosis were ipsilateral breast tumor recurrence (IBTR) as the first event within 10 years of randomization and breast cancer death (BCD) within 15 years of randomization. For radiotherapy efficacy, the interaction between WRAP53 and radiotherapy was assessed, in relation to IBTR within 10 years. We hypothesized that low levels of WRAP53 are associated with a worse prognosis and resistance to radiotherapy. Two different WRAP53 antibodies were evaluated, C1 and C2. C1 has been used in several previous projects [11, 13, 15, 20] whereas C2 is newer and not as well‐studied [5, 12]. The hypothesis was that the immunohistochemical staining using the two antibodies would correlate but since they bind to different parts of the protein the correlation would not be perfect. Moreover, we investigated whether radiotherapy treatment led to the enrichment of tumor cells with low levels of WRAP53 protein in the subsequent local recurrence, compared with the paired primary tumor. Furthermore, using gene expression data, we evaluated transcriptome and pathway differences between tumors with high and low WRAP53 protein levels.

## 2 Materials and methods

### 2.1 SweBCG91RT—patients and study design

**Fig. 1:**
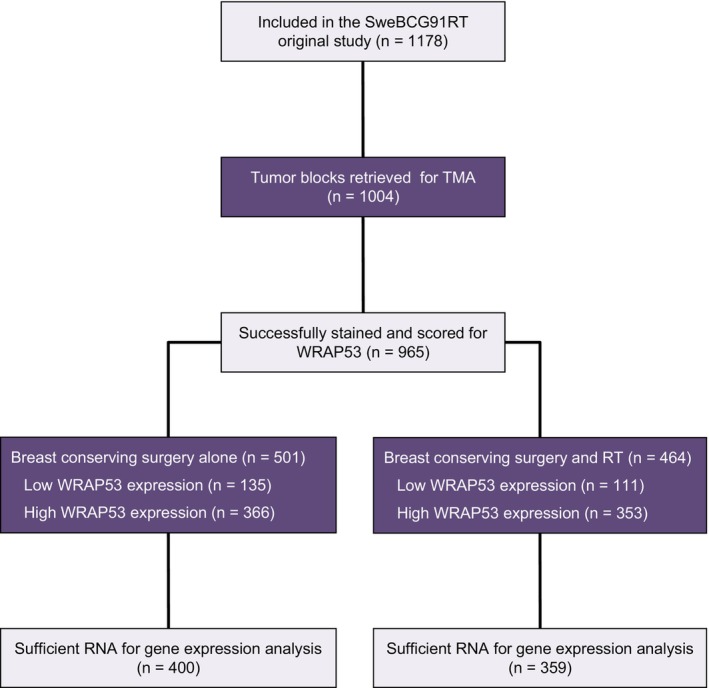
CONSORT diagram over the study design and number of tumors available for analysis in each group, as well as number of tumors included in gene expression analysis. TMA, tissue microarray; RT, radiotherapy.

The study design of SweBCG91RT has been previously described [17], as well as the 15‐year follow‐up [18]. In summary, 1178 patients with T1‐2N0M0 primary, invasive breast cancer were randomized to postoperative radiotherapy or not after breast‐conserving surgery, between 1991 and 1997 in Sweden. All tumors were radically resected. Radiotherapy was given to the whole remaining breast parenchyma, in total 48–50 Gy over 24–27 fractions [18]. Apart from radiotherapy, adjuvant treatment was given only to 9% of all patients. During follow‐up, medical records were revised for cases of recurrent cancer. The Swedish population registry and the Cause of death registry were used for information on vital status and causes of death. Both registries have very high completeness and accuracy. A tissue microarray (TMA) containing cores of formalin‐fixed paraffin‐embedded material from available tumors (primary tumors *n* = 1004; IBTR *n* = 141) was constructed later [19]. Representative areas of the tumor tissue were marked by a board‐certified pathologist on hematoxylin and eosin sections of the tumor block. The TMA contains two 1.0‐mm cores from the centre of this area. Previously, new immunohistochemical stainings for the estrogen receptor (ER), progesterone receptor (PgR), and proliferation marker Ki‐67 have been performed. For human epidermal growth factor receptor 2 (HER2) a combination of immunohistochemical staining and *in situ* hybridization was used. Cancer subtypes were assigned according to the guidelines of the St Gallen International Breast Cancer conference (2013) Expert Panel [21], but as a result of group sizes, the luminal and nonluminal HER2‐positive tumors were combined into one group. Subtypes and group sizes for primary tumors were thus 555 luminal A‐like (ER+, PgR+, HER2−, Ki‐67 low), 259 luminal B‐like (PgR− and/or Ki67 high, ER+, HER2−), 64 HER2+ (HER2+, any ER or PgR status, any Ki‐67), 81 triple negative (ER−, PgR−, HER2−, any Ki‐67), and 45 with missing data. Out of all 1004 primary tumors included in the TMA, 765 were available and had sufficient RNA for gene expression analysis (Fig. 1). Gene expression data acquisition has been described previously [22]. Briefly, RNA was extracted from 1.5 mm tumor tissue punches employing the RNeasy FFPE kit (Qiagen, Hilden, Germany). cDNA was amplified using the Ovation FFPE WTA system (NuGEN, San Carlos, CA, USA), fragmented, and labeled using the Encore Biotin Module (NuGEN). It was hybridized to GeneChip Human Exon 1.0 ST Arrays. Data were normalized by the manufacturing company (Thermo Fisher Scientific, South San Francisco, CA, USA) through single array normalization upon delivery [23]. Tumors excluded from the TMA were marginally smaller, and for RNA expression analysis also slightly more likely to be luminal A‐like and of histologic grade 1 [19, 22]. For the current study, radiotherapy treatment status was classified according to the given treatment and not intention‐to‐treat. Out of 1004 patients included in the TMA, 23 randomized to postoperative radiotherapy did not receive radiotherapy and three patients randomized to control were given radiotherapy. The study was prepared in accordance with the principles of the Helsinki Declaration of 1964. All patients gave informed oral consent in the original trial, which was approved by Regional Ethical Review Board at Lund University (approval number 80/90). The follow‐up and the TMA creation were also approved by the regional ethical review board (approval numbers 2010/127 and 2015/548, respectively).

### 2.2 Immunohistochemical staining and scoring

Tissue microarray sections were stained with two primary WRAP53 antibodies [15, 24] targeting different sections of the protein: C1 (amino acids 483–496, polyclonal, dilution 1 : 45, Innovagen, Lund, Sweden) and C2 (amino acids 535–548, polyclonal, dilution 1 : 300, Innovagen), both commercially available. Immunohistochemistry staining was performed on Autostainer Plus instrument (DAKO, Agilent Technologies, Santa Clara, CA, USA) and visualized by using Envision Flex DAB Detection kit (Agilent Technologies). For both WRAP53 antibodies the core with the highest fraction of stained tumor cells was used [15]. The correlation between the two cores was strong (Spearman's ρ = 0.7). Cores with fewer than 50 tumor cells were excluded from the analysis [15]. Scoring was performed by a breast pathologist. There is no standardized method to immunohistochemically assess WRAP53 protein levels. Previous studies have used different scoring methods with the percentage of stained cells, intensity of staining, and the subcellular localization of the protein [11, 12, 15]. To maximize the information from the immunohistochemical scoring, a combination of all was used. The fraction of tumor cells with positive nuclear staining was categorized as 0 (0%), 1 (1–9%), 2 (10–50%), 3 (51–75%), or 4 (> 75%). The staining intensity was classified as 0 (Negative), 1 (Weak), 2 (Moderate), or 3 (Strong). A nuclear histoscore (the fraction score * the intensity score) was used (histoscore 0–12). Cytoplasmic staining was also assessed [15] but in the following text protein levels refer to nuclear staining if not stated otherwise. To create two groups of low and high nuclear WRAP53 protein levels, a cutoff as close to the 25^th^ percentile as possible was used [22], hypothesized to represent the approximately 25% of patients with node‐negative breast cancer developing locoregionally recurrent disease within 10 years without radiotherapy [2]. Based on staining results, a cutoff at histoscore 6 was applied (< 6 low, > 6 high), correlating to the 25^th^ percentile for C1‐antibody and the 28^th^ percentile for C2‐antibody. Separation at the 25^th^ percentile was also employed for dichotomizing *WRAP53* RNA levels when needed.

### 2.3 Statistics

Statistical analysis was mainly performed using stata (v.17, College Station, TX, USA). r (v.3.6.1, Vienna, Austria) was employed for RNA expression processing and analysis. Spearman's rank correlation was used to test the correlation between WRAP53 histoscores (0–12) using the two antibodies. Levels of WRAP53 protein and *WRAP53* RNA were correlated with clinicopathological variables using the Pearson's chi‐squared test, Fisher's exact test, independent‐sample *t*‐test, and Wilcoxon rank‐sum test where appropriate. Competing‐risk regression according to the method of Fine and Gray [25] was performed for IBTR (with other recurrences and death as competing risks) and BCD (with death from other causes as competing risk). The IBTR analysis was restricted to the first 10 years after randomization to reflect true recurrences, the effect of radiotherapy and reduce the number of new, and ipsilateral breast tumors. For BCD, a cutoff at 15 years was selected to ensure that the majority of the patients were included in the analysis and to decrease bias depending on the time of inclusion. Separate analyses were made for WRAP53 protein levels and *WRAP53* RNA expression, as well as radiotherapy, adjuvant therapy, subtype, histologic grade, age, and tumor size. In multivariable analysis, all significant variables, tumor size, and subtype were kept. The multivariable IBTR analysis included WRAP53, radiotherapy, adjuvant therapy, subtype, histologic grade, tumor size, and age. BCD analysis included WRAP53, subtype, histologic grade, and tumor size. Subhazard ratios (SHRs) and 95% confidence intervals (95% CIs) are presented. To test WRAP53 as a marker predictive of radiotherapy, efficacy interaction terms between WRAP53 levels (protein and RNA) and radiotherapy were introduced in the IBTR analyses. The proportional hazard assumption was tested using time interaction with all covariates. It was violated for *WRAP53* RNA expression in IBTR and BCD analysis. The SHRs presented for *WRAP53* RNA expression must thus be interpreted as mean effects over the entire period. Cumulative incidence functions with competing risks were created with the user‐written command stcompet.ado [26]. Differential expression testing in WRAP53 protein levels between 90 paired primary tumors and IBTRs was performed depending on radiotherapy treatment using the Wilcoxon rank‐sum test. In the RNA expression dataset, linear models and Gene Set Enrichment Analysis (GSEA) [27] were employed to derive differential expression and pathway enrichments, respectively. Data were matched against patient data to derive a list of 16 768 genes across 759 samples. Differential expression between high and low WRAP53 protein levels was assessed by linear models using limma, and the resulting *P*‐values were adjusted using the Benjamini–Hochberg method. Pathway enrichment analyses were performed using GSEA. Data were queried against the Hallmarks database (v 5.2) [28], permutation type was set to gene set, weighted scoring was enabled, and *t*‐test was selected as metric. All other parameters were kept at default settings. The pathway significance cutoff was set to FDR < 0.25. In all analyses, a *P*‐value < 0.05 was considered significant.

## 3 Results

### 3.1 Protein levels and RNA expression

**Fig. 2:**
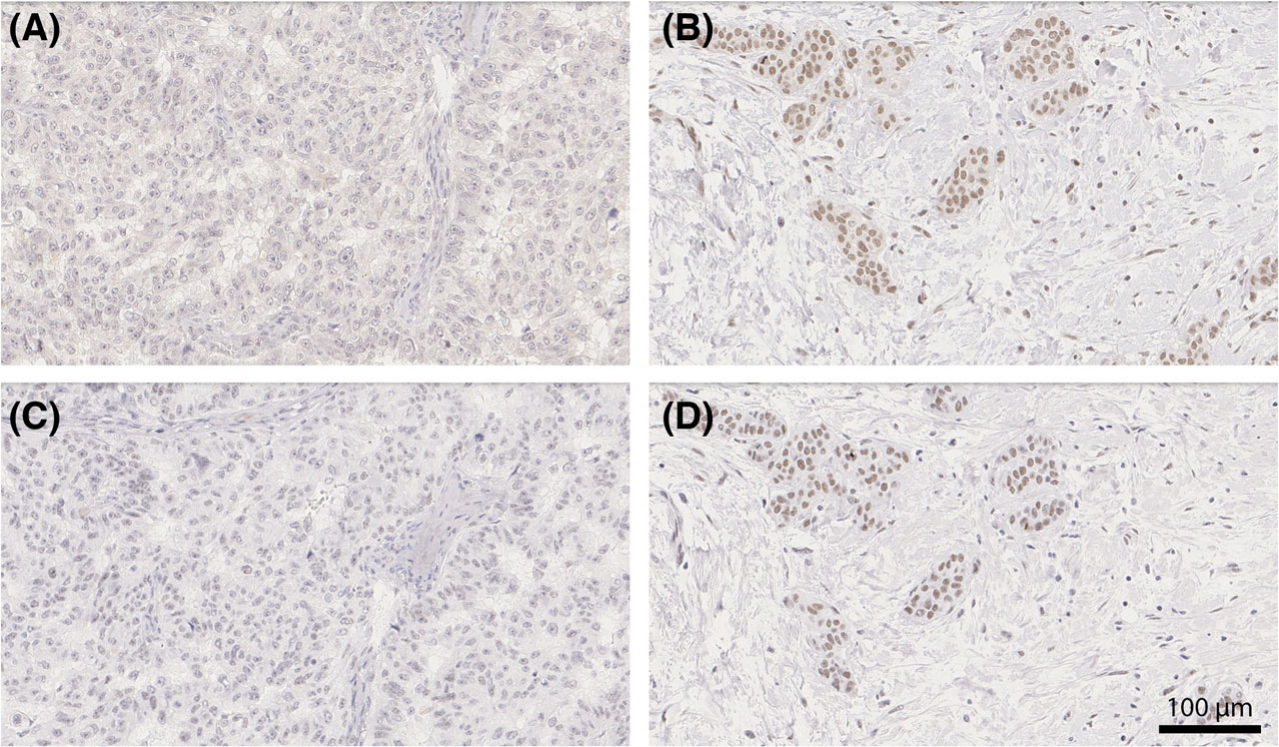
(A–D) Immunohistochemical staining for WRAP53 (C1‐ and C2‐antibody) and their respective categories based on histoscore (< 6 low, > 6 high). (A) Low nuclear staining score (C1). (B) High nuclear staining score (C1). (C) Low nuclear staining score (C2). (D) High nuclear staining score (C2). Scale bar representing 100 μm.

In total, 1004 primary tumors were included in the TMA and 965 were successfully stained and scored for WRAP53 protein using two WRAP53 antibodies (C1 and C2, Fig. 2). Tumors were classified using a histoscore as having either low levels of WRAP53 protein (consistent in 246 and 270 primary tumors using C1‐ and C2‐antibody, respectively) or high levels (in 719 and 695 tumors, respectively). There was a weak correlation for the histoscores (Spearman's ρ = 0.27, *P* < 0.001; Fig. S1). Cytoplasmic staining was more common using the C1‐antibody than the C2‐antibody. Of all tumors successfully stained and scored for WRAP53, 759 had sufficient material for gene expression analysis of which 190 were classified as having low *WRAP53* RNA and 569 having high RNA levels. Scoring concordance and discrepancies are presented in Table S1. There were no significant associations between protein levels of WRAP53 using C1‐ or C2‐antibody and *WRAP53* RNA expression (*P* = 0.20 and *P* = 0.79, respectively, Fig. S2).

### 3.2 Association of WRAP53 to clinicopathological variables

The associations between WRAP53 and known important clinicopathological variables were investigated and were presented in Table 1 for protein levels using C1‐antibody and in Table S2 for C2‐antibody and RNA levels. Low levels of WRAP53 protein, according to C1‐antibody, were associated with factors indicating worse prognosis (larger tumor size, higher TNM stage, higher frequency of HER2‐positive and triple‐negative subtype, and histologic grade III). Adjuvant therapy was also more common among patients with tumors with low WRAP53 protein levels. Low protein levels according to C2‐antibody showed the same tendency but not *WRAP53* RNA levels. Thus, clinicopathological variables associated with worse prognosis are more common among tumors with low levels of WRAP53 protein.

**Table 1:** Patient and tumor characteristics in relation to nuclear WRAP53 protein levels (C1‐antibody). *P*‐values < 0.05 in bold. SD, standard deviation; Q1–Q3, interquartile range.

	WRAP53 protein (C1)		
	Low (*n* = 246)	High (*n* = 719)	*P*‐value
Age, years			0.085^a^
Mean (SD)	57.7 (9.96)	58.9 (9.22)	
Radiotherapy			0.28^b^
None	135 (55%)	366 (51%)	
Radiotherapy	111 (45%)	353 (49%)	
Adjuvant therapy			**< 0.001** ^c^
None	210 (85%)	676 (94%)	
Endocrine therapy	27 (11%)	35 (5%)	
Chemotherapy	3 (1%)	7 (1%)	
Both	6 (2%)	1 (0%)	
Tumor size, mm, missing 6			**< 0.001** ^d^
Median (Q1–Q3)	15 (11–19)	12 (9–15)	
TNM stage			**< 0.001** ^b^
T1aN0M0	4 (2%)	25 (3%)	
T1bN0M0	55 (23%)	285 (40%)	
T1cN0M0	144 (60%)	362 (50%)	
T2N0M0	39 (16%)	45 (6%)	
Missing data	4	2	
Subtype			**< 0.001** ^b^
Luminal A‐like	108 (45%)	438 (63%)	
Luminal B‐like	64 (26%)	192 (27%)	
HER2‐positive	27 (11%)	35 (5%)	
Triple‐negative	43 (18%)	35 (5%)	
Missing data	4	19
Histologic grade			**< 0.001** ^b^
Grade I	12 (5%)	130 (19%)	
Grade II	114 (48%)	446 (64%)	
Grade III	112 (47%)	122 (17%)	
Missing data	8	21	

a Independent‐sample *t*‐test.

b Chi‐square test.

c Fisher's exact test.

d Wilcoxon rank‐sum test.

### 3.3 Prognosis for ipsilateral breast tumor recurrence and breast cancer death

In the next step, WRAP53 protein and *WRAP53* RNA levels were evaluated as prognostic markers for IBTR within 10 years and BCD within 15 years in uni‐ and multivariable competing‐risk regression analysis. The median follow‐up was 18 years for all patients and 20 years for patients alive at the last follow‐up, respectively. Of the three markers, low protein levels using C1‐antibody showed the highest association with IBTR with an SHR of 1.87 (95% CI 1.33–2.63, Table S3), in univariable analysis and SHR 1.76 (95% CI 1.10–2.79, Table 2) in multivariable analysis. Low protein levels using C1‐antibody were also associated with a significantly increased incidence of BCD in both univariable analyses (SHR 2.17, 95% CI 1.52–3.10, Fig. S3) and multivariable analysis (SHR 1.55, 95% CI 1.02–2.38, Table 2). WRAP53 protein levels using C2‐antibody and *WRAP53* RNA levels were not prognostic for IBTR and BCD (Table 2 and Table S3). Absolute events stratified by WRAP53 levels are presented in Table S4. A further division of WRAP53 protein levels into three groups based on histoscores (low, intermediate, and high) in relation to IBTR and BCD is presented in Table S5. No dose–response relationship was found for IBTR as tumors with intermediate protein expression (C1) displayed the lowest SHR. For BCD, increasing protein levels correlated with decreasing SHRs, however, not significant. Cytoplasmic staining did not add any valuable information or change the multivariable analysis significantly (data not shown). We conclude that WRAP53 low protein levels in comparison with high protein levels, using C1‐antibody, are prognostic for IBTR and BCD.

**Table 2:** Multivariable competing‐risk regression depending on nuclear WRAP53 (C1‐ and C2‐antibody) and *WRAP53* RNA levels for IBTR within 10 years and BCD within 15 years. The presented effect of RT is in tumors with high WRAP53. The SHR for RT effect in tumors with low WRAP53 is calculated as main effect of RT in WRAP53 high * interaction effect. In IBTR analysis also adjusting for adjuvant therapy, subtype, histologic grade, tumor size, and age. *P*‐values < 0.05 in bold. In BCD analysis also adjusting for subtype, histologic grade, and tumor size. BCD, breast cancer death; CI, confidence interval; IBTR, ipsilateral breast tumor recurrence; ref, reference; RT, radiotherapy; SHR, subhazard ratios.

	WRAP53 protein (C1)		WRAP53 protein (C2)		*WRAP53* RNA	
	SHR (95% CI)	*P*‐value	SHR (95% CI)	*P*‐value	SHR (95% CI)	*P*‐value
IBTR						
WRAP53						
High (ref)	1		1		1	
Low	1.76 (1.10–2.79)	**0.017**	1.21 (0.76–1.92)	0.43	0.91 (0.54–1.52)	0.71
RT						
No RT (ref)	1		1		1	
RT	0.39 (0.24–0.62)	**< 0.001**	0.35 (0.22–0.55)	**< 0.001**	0.33 (0.19–0.55)	**< 0.001**
Interaction						
WRAP53 – RT	1.13 (0.51–2.50)	0.76	1.47 (0.66–3.30)	0.35	2.66 (1.13–6.24)	**0.024**
BCD						
WRAP53						
High (ref)	1		1		1	
Low	1.55 (1.02–2.38)	**0.042**	1.02 (0.69–1.51)	0.94	1.12 (0.72–1.74)	0.61

### 3.4 Prediction of radiotherapy efficacy

**Fig. 3:**
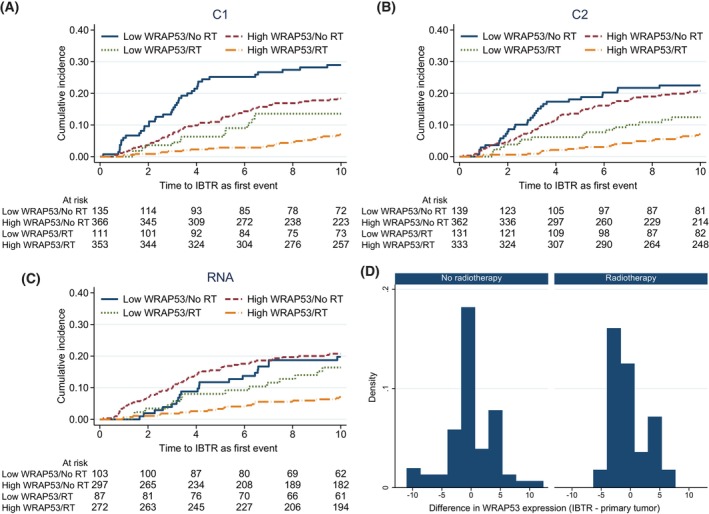
(A–D) Cumulative incidence of ipsilateral breast tumor recurrence (IBTR) in the presence of competing risks depending on WRAP53 and radiotherapy (RT) using protein levels from C1‐antibody (A), protein levels from C2‐antibody (B), and RNA levels of *WRAP53* (C). Time in years. Difference in nuclear WRAP53 protein levels (C1‐antibody) in primary tumor compared with IBTR, depending on RT. Negative values correspond to reduction in WRAP53 levels in IBTR compared with primary tumor (D).

Further, the evaluation of WRAP53 as a marker predictive of radiotherapy efficacy was performed. There was a significant interaction between RNA expression of WRAP53 and radiotherapy efficacy (*P* = 0.024). Radiotherapy greatly reduced the incidence of IBTR in tumors with high *WRAP53* RNA expression (SHR 0.33, 95% CI 0.19–0.55, Table 2), but not in tumors with low levels of *WRAP53* RNA (SHR 0.87, 95% CI 0.44–1.72). The results are also illustrated in Fig. 3A–C. When the further division of RNA levels into quartiles was made, no dose–response relationship was found (data not shown). Tumors with low WRAP53 protein levels (C2) had a slightly reduced effect of radiotherapy (SHR 0.51, 95% CI 0.27–0.98) compared to tumors with high WRAP53 (SHR 0.35, 95% CI 0.22–0.55) but with little evidence for an interaction (*P* = 0.35). Radiotherapy efficiently reduced the incidence of IBTR in both patients with high levels of WRAP53 protein using C1‐antibody (SHR 0.39, 95% CI 0.24–0.62, Table 2) and patients with low levels of WRAP53 protein (SHR 0.44, 95% CI 0.23–0.83). Thus, there was no evidence for an interaction between WRAP53 protein levels (C1) and radiotherapy (*P* = 0.76). Our results indicate that low levels of *WRAP53* RNA are predictive of radiotherapy resistance.

### 3.5 WRAP53 in primary tumors and IBTRs

To study whether tumor cells with low WRAP53, as a consequence of radioresistance, are enriched in locally recurring cancer after radiotherapy, WRAP53 protein levels were compared between primary tumors and subsequent IBTRs. Using the histoscores (0–12) based on C1‐antibody, 10 tumors (42%) showed reduced protein levels in the recurrence after radiotherapy compared with the primary tumor, seven (29%) did not change and seven (29%) had higher WRAP53 levels in the recurrence after radiotherapy. In the nontreated group, the corresponding numbers were 19 (29%), 25 (38%), and 22 (33%). In the Mann–Whitney *U* test, comparing the difference in WRAP53 protein levels between recurrence and primary tumor depending on radiotherapy treatment, there was a weak tendency towards local recurrences having lower WRAP53 levels after radiotherapy than if untreated, but little evidence was found (*P* = 0.35, Fig. 3D). Thus, little evidence for an enrichment of low WRAP53 levels in locally recurrent cancer after radiotherapy was found.

### 3.6 Differently expressed genes and pathways

**Fig. 4:**
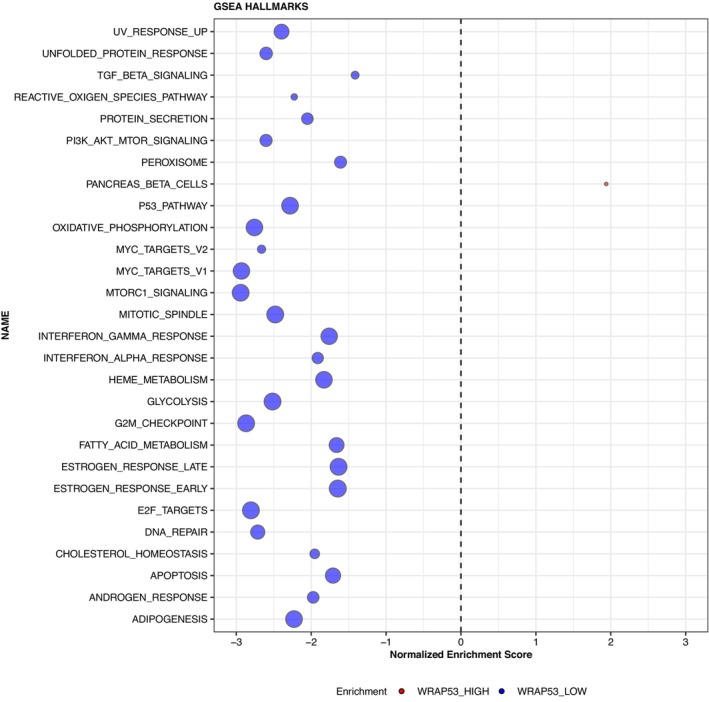
Gene set enrichment analysis (GSEA, Hallmarks database) depending on nuclear WRAP53 protein levels (C1‐antibody). Blue dots (left‐hand side) represent pathways enriched in tumors with low WRAP53 protein. Red dot (right‐hand side) represents the one pathway enriched in tumors with high WRAP53 protein (Pancreas beta cell pathway). The size of the dot corresponds to the size of the gene set.

Differential expression analysis was performed to investigate the cellular processes that could contribute to the impaired prognosis and radiotherapy resistance in tumors with low protein levels of WRAP53. The analysis showed that 8100 genes were differentially expressed between tumors with low and high WRAP53 protein levels (C1). Of these, 5166 genes were enriched in tumors with high WRAP53 (e.g., ADH1B, FABP4, and FOSB) and 2934 in tumors with low WRAP53 (e.g., RPN2, ARF1, and CALR). GSEA showed that networks enriched in tumors with low WRAP53 related to the p53 pathway, DNA repair pathway, and apoptosis pathway among others (Fig. 4). Only the pancreas beta cell pathway was enriched in tumors with high WRAP53. The discrepancy between GSEA according to C1‐ and C2‐antibody is presented in Fig. S4. In summary, gene pathways enriched in tumors with low WRAP53 according to both antibodies were related to DNA damage, proliferation, and inflammatory response. The expression of *WRAP53* gene itself did not correlate well (Spearman's ρ > 0.5) with any other gene. A list of the top 20 genes correlated with *WRAP53* is presented in Table S6. Thus, the DNA repair, p53, and apoptosis pathways among several other cellular processes are involved in tumors with low WRAP53 protein levels.

## 4 Discussion

We have shown that protein levels of WRAP53 and *WRAP53* RNA expression are associated with prognosis in breast cancer and resistance to radiotherapy. Low protein levels, using C1‐antibody, were associated with a significantly increased risk for both IBTR and BCD. C2‐antibody and *WRAP53* RNA levels were not prognostic for IBTR and BCD. Low levels of *WRAP53* RNA were predictive of radiotherapy efficacy, with almost a three‐fold increased incidence of IBTR despite radiotherapy. Tumors with low protein levels according to C2‐antibody had a tendency towards reduced radiotherapy efficacy.

To our knowledge, the association between low levels of nuclear WRAP53 and increased risk of locally recurrent cancer has not been shown previously in breast cancer. Thus, the finding of WRAP53 as a prognostic marker for IBTR is new. Previous studies have found that low nuclear levels of WRAP53 correlate with an increased mortality, which is in line with our findings. In head and neck cancer, the association was for overall survival [11], and in breast cancer for breast cancer‐specific mortality [15]. In ovarian cancer, low levels of both *WRAP53* mRNA and WRAP53 nuclear staining correlated with reduced survival [12]. We were unable to find such an association as *WRAP53* RNA levels did not correlate with neither the nuclear staining of WRAP53 nor prognosis.

Expression of *WRAP53* was found predictive of response to radiotherapy. This is an interesting finding and is in accordance with our hypothesis that tumors with low WRAP53 expression are less sensitive to radiotherapy treatment. Low levels of *WRAP53* RNA corresponded to almost a three‐fold higher risk of IBTR within 10 years after radiotherapy compared to the 75% of tumors with higher levels of *WRAP53* RNA. No dose–response relationship could be found when further subdivision of RNA levels was made. This could be due to small group sizes or that only the tumors with the least *WRAP53* expression are resistant to radiotherapy. Previous research on WRAP53 and radiotherapy resistance has shown conflicting results. Low staining of WRAP53 was more common in head and neck cancer patients classified as nonresponders to radiotherapy [11], which is in line with our findings although on the protein level. In rectal cancer, it has also been shown that the protein levels of WRAP53 were reduced by preoperative radiotherapy [13], suggesting that WRAP53 negativity is enriched by radiotherapy. In our limited material on primary tumors and subsequent local recurrences after radiotherapy such a tendency was found but was insignificant. Conversely, in a report presenting the predictive value of several biomarkers in rectal cancer biopsy samples patients with high nuclear WRAP53 did not seem to benefit from radiotherapy [20]. This is opposed to our finding but lacks an interaction analysis. On a cellular level, studies have shown that cells overexpressing WRAP53 repair DNA damage more efficiently after irradiation and have reduced pro‐apoptotic signaling [5]. Thus, the cancer cell is potentially dependent on an upregulated DNA damage response before WRAP53 expression is lost. Loss of WRAP53 activity is then favorable for the tumor cell and likely reflects the survival of DNA repair‐deficient and genetically unstable cells that upon clonal expansion drives tumor progression. More research is needed on the relationship between WRAP53 and radiotherapy resistance.

SweBCG91RT is a large randomized controlled trial with a very long follow‐up of patients with early‐stage breast cancer, where patients were randomized to postoperative radiotherapy or not. The majority of the tumors were included in both a tissue microarray, allowing for the staining of new markers, and RNA expression analysis. This makes SweBCG91RT an ideal trial for evaluating radiotherapy response in relation to new markers. Potential limitations of this study include that the cohort lacks patients with more advanced stages of breast cancer. However, the patients included represents the majority of women in a modern breast cancer cohort making the results generalizable. Second, few patients were recommended systemic adjuvant treatment at the time, which a majority would have been today. Such treatment would have decreased the risk of recurrences, making our results harder to translate to a modern setting. However, this is also a strength since it gives a unique opportunity to study the effect of radiotherapy without confounding other types of treatment. It is not known how systemic therapy would affect the specific functions of WRAP53. It appears likely that this protein would be activated and participate in the repair of DNA damage caused by various genotoxic agents, as WRAP53 has been shown to be phosphorylated and activated by ATM following various stress conditions [6]. Cells that lack WRAP53 could be more sensitive to DNA damage caused by chemotherapy or, as with radiotherapy, be more resistant to it. The transcription of WRAP53 could potentially be activated by hormones and anti‐estrogen treatment leading to lesser expression of WRAP53. Thus, further studies with modern adjuvant therapy are needed to decide how our results could be implemented in clinical practice.

The diverging results between the three WRAP53 expression evaluation methods (C1, C2, and RNA) in regards of prognostic and radiotherapy efficacy predictive abilities indicate that this is a complex subject that calls for further research. The discrepancy is reflected in the weak (or nonexisting) correlation between the results from the different measurements. For the two antibodies, it is likely caused by variation in antibody specificity. C1 and C2 bind to separate sections of the protein and thus potentially different isoforms of the protein. These isoforms could be differently affected by cellular processes, which could contribute to the discrepancy in staining patterns and correlation to outcome. Other contributing factors could be intratumoral heterogeneity and slightly different sections of the tumor being used in the two cores for each antibody and for the two antibodies. Evaluating both cores from each tumor together, rather than separately and choosing the one with the highest fraction of stained tumor cells, would have decreased the effect of a potential intratumoral heterogeneity. However, the correlation of histoscores for the tumor pairs was strong and intratumoral heterogeneity is not considered to have a large impact on the results. As for the discrepancy between protein and RNA levels, the two diagnostics methods differ fundamentally. In gene expression analysis, serial whole sections from formalin‐fixed paraffin‐embedded material are analyzed, incorporating also the *WRAP53* RNA from noncancerous tissue cells in the tumor. On the protein level, pathologists make an assessment only on tumor cells, not the whole slide. Further, the two methods represent different stages of WRAP53 expression (protein and RNA) and biological variations such as post‐translational regulation or the rate of degradation of RNA and protein could increase discrepancy. Additionally, both methods could be affected by slightly differing specificity in binding to their corresponding protein and mRNA. Some of the probes in the GeneChip bind to *WRAP53*α‐ and *WRAP53γ*‐transcripts, as well as to the transcript of interest, *WRAP53β*. However, the α‐ and *γ*‐transcripts contribute to a very small portion of the total RNA amount. For these reasons, it would be interesting to quantify WRAP53 protein using mass‐spectrometry and correlate with RNA levels and immunohistochemical staining, using different antibodies.

In the gene set enrichment analysis, DNA damage response‐related gene pathways, among others, were enriched in tumors with low WRAP53 protein levels. This is in line with the hypothesis that as the tumor progresses, the DNA damage response is activated. Loss of WRAP53 in this situation allows the escape of damaged cancer cells and the formation of even more malignant clones. A tumor cell would likely not be advantaged by complete loss of DNA damage response since that would generate such a large amount of mutations and DNA breaks that the cell was unable to function. However, some reduction in DNA repair function is favorable as this promotes the gain of new oncogenic or loss of tumor suppressor genes. The majority of the differently expressed genes were enriched in tumors with high WRAP53, but this did not correspond to any pathways. This lack of enriched pathways can be due to a large heterogeneity among tumors with high WRAP53 levels.

## 5 Conclusion

We have shown that immunohistochemical assessment of WRAP53 protein using C1‐antibody is prognostic in early‐stage breast cancer, as low nuclear levels were associated with a higher incidence of locally recurrent cancer and death from breast cancer. Low levels of *WRAP53* RNA were associated with resistance to radiotherapy. More knowledge about WRAP53 and radiotherapy efficacy is needed, as well as further studies investigating the relationship between nuclear protein and RNA expression of WRAP53.

## Author contributions

ME and EN involved in all parts. GP and LT involved in the acquisition and interpretation of TMA data. TDM involved in the analysis and interpretation of gene expression data. P‐OB, AST, EH, PK, MF, and FK involved in the design of study and interpretation of data. All authors involved in writing and drafting the manuscript and the approval of the final version.

## Conflict of interest

Erik Holmberg and Per Karlsson have research contracts with PFS genomics and Prelude DX and patents pending, not related to the current study.

### Peer review

The peer review history for this article is available at https://www.webofscience.com/api/gateway/wos/peer‐review/10.1002/1878‐0261.13426.

## Supporting information


**Fig. S1.** Scatter plot with jitter of the nuclear staining results from the two antibodies.
**Fig. S2.** Violin plot of expression of *WRAP53* RNA levels in relation to nuclear WRAP53 protein.
**Fig. S3.** Cumulative incidence functions of time to breast cancer death in 15 years depending on WRAP53 levels (C1‐ and C2‐antibody) and *WRAP53* RNA expression.
**Fig. S4.** Scatter plot over gene pathways (Hallmarks database) enriched in tumors depending on WRAP53 protein levels (low and high) according to C1‐ and C2‐antibody.
**Table S1.** Concordance of WRAP53 levels using C1‐antibody, C2‐antibody, and *WRAP53* RNA.
**Table S2.** Patient and tumor characteristics in relation to nuclear WRAP53 protein levels (C2‐antibody) and *WRAP53* RNA levels.
**Table S3.** Univariable competing‐risk regression depending on nuclear WRAP53 (C1‐ and C2‐antibody) and RNA levels for IBTR within 10 years and BCD within 15 years.
**Table S4.** Absolute events in IBTR‐ and BCD‐competing‐risk analysis stratified by WRAP53 levels presented as total number (% of all).
**Table S5.** Multivariable competing‐risk regression depending on three levels of nuclear WRAP53 (C1‐ and C2‐antibody) for IBTR within 10 years and BCD within 15 years.
**Table S6.** Top 20 genes significantly correlating with *WRAP53* RNA expression.

## Data Availability

Gene expression data are available at Gene Expression Omnibus, GSE119295. Patient information and follow‐up data cannot be disclosed, due to restrictions in patient confidentiality and the ethical approval.
