# Supplementary material for: Low levels of WRAP53 predict decreased efficacy of radiotherapy and are prognostic for local recurrence and death from breast cancer: a long‐term follow‐up of the SweBCG91RT randomized trial
Source: Mol Oncol. 2023 Apr 19;17(10):2029–40. doi: 10.1002/1878-0261.13426 (PMC10552889; doi:10.1002/1878-0261.13426)
Supplement: Supplementary file 1 — Fig. S1. Scatter plot with jitter of the nuclear staining results from the two antibodies. Fig. S2. Violin plot of expression of WRAP53 RNA levels in relation to nuclear WRAP53 protein. Fig. S3. Cumulative incidence functions of time to breast cancer death in 15 years depending on WRAP53 levels (C1‐ and C2‐antibody) and WRAP53 RNA expression. Fig. S4. Scatter plot over gene pathways (Hallmarks database) enriched in tumors depending on WRAP53 protein levels (low and high) according to C1‐ and C2‐antibody. Table S1. Concordance of WRAP53 levels using C1‐antibody, C2‐antibody, and WRAP53 RNA. Table S2. Patient and tumor characteristics in relation to nuclear WRAP53 protein levels (C2‐antibody) and WRAP53 RNA levels. Table S3. Univariable competing‐risk regression depending on nuclear WRAP53 (C1‐ and C2‐antibody) and RNA levels for IBTR within 10 years and BCD within 15 years. Table S4. Absolute events in IBTR‐ and BCD‐competing‐risk analysis stratified by WRAP53 levels presented as total number (% of all). Table S5. Multivariable competing‐risk regression depending on three levels of nuclear WRAP53 (C1‐ and C2‐antibody) for IBTR within 10 years and BCD within 15 years. Table S6. Top 20 genes significantly correlating with WRAP53 RNA expression. [file MOL2-17-2029-s001.pdf]

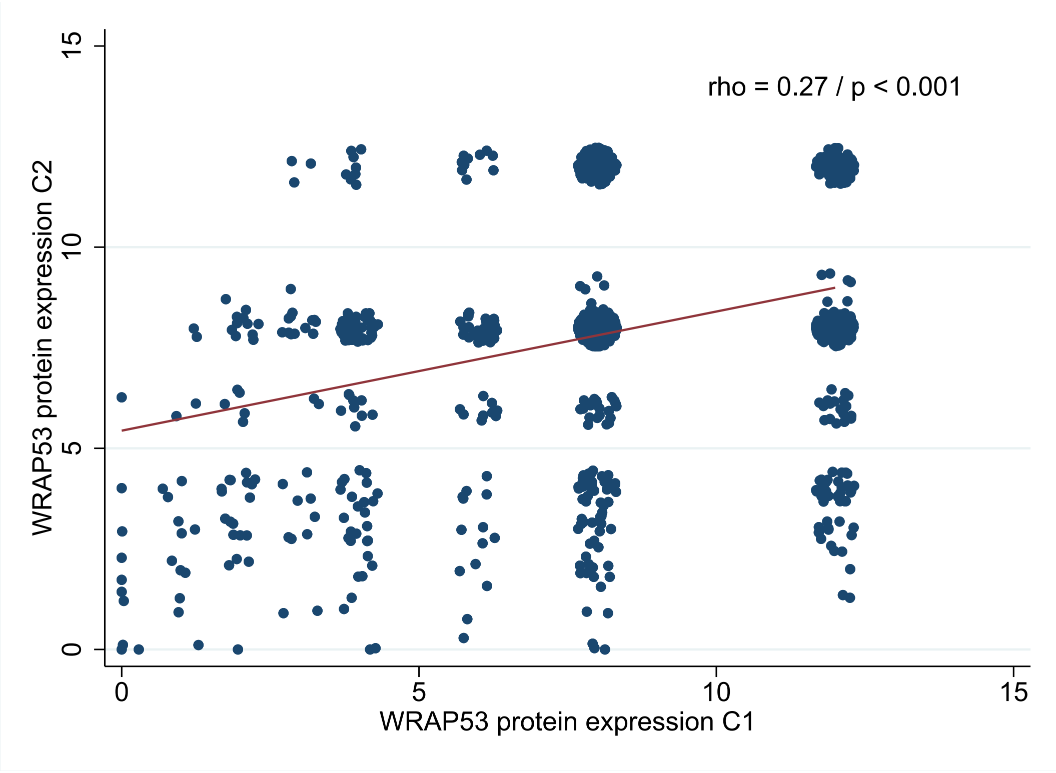


<u>Figure S1.</u> Scatter plot with jitter of the nuclear staining results from the two antibodies used (C1 and C2). rho- and p-value from Spearman's rank test.

Table S1. Concordance of WRAP53 levels using C1-antibody, C2-antibody and *WRAP53* RNA.

| | | **C1 (965)** | | **C2 (965)** | | **RNA (759)** | |
|---|---|---|---|---|---|---|---|
| | | Low | High | Low | High | Low | High |
| **C1 (965)** | Low | 246 | - | 120 | 126 | 59 | 148 |
| | High | - | 719 | 150 | 569 | 131 | 421 |
| **C2 (965)** | Low | 120 | 150 | 270 | - | 59 | 140 |
| | High | 126 | 569 | - | 695 | 131 | 429 |
| **RNA (759)** | Low | 59 | 131 | 59 | 131 | 190 | - |
| | High | 148 | 421 | 140 | 429 | - | 569 |

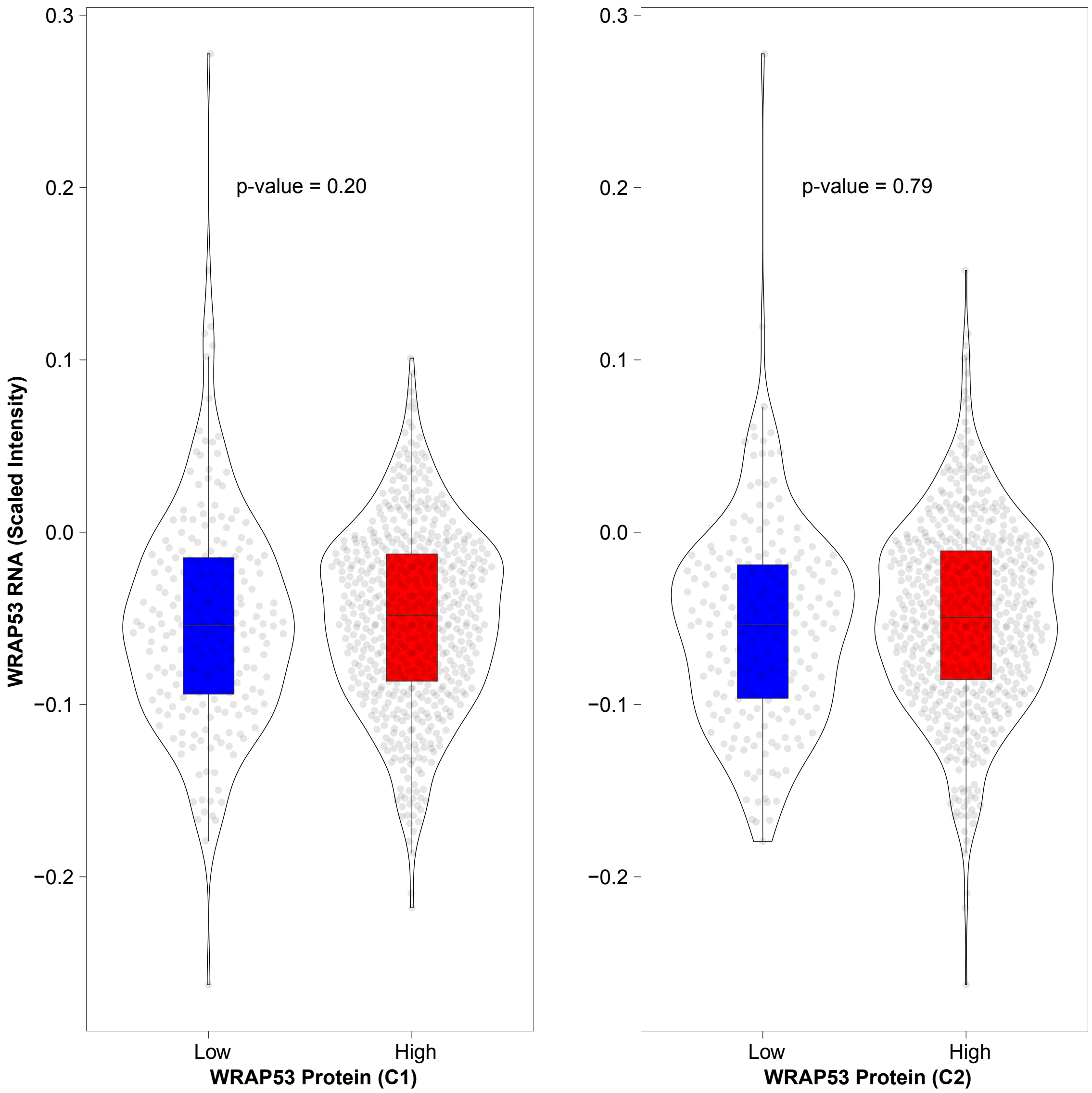


<u>Figure S2.</u> Violin plot of expression of *WRAP53* RNA levels in relation to nuclear WRAP53 protein (C1- and C2-antibody, respectively). p-values from LIMMA analysis.

Table S2. Patient and tumor characteristics in relation to nuclear WRAP53 protein levels (C2-antibody) and *WRAP53* RNA levels. Missing values not included in tests.

| | **WRAP53 protein (C2)** | | | ***WRAP53* RNA** | | |
|---|---|---|---|---|---|---|
| | Low (n = 270) | High (n = 695) | p-value | Low (n = 190) | High (n = 759) | p-value |
| **Age**, years | | | 0.046 [a] | | | 0.62 [a] |
| Mean (SD) | 59.6 (9.72) | 58.2 (9.24) | | 58.2 (9.30) | 58.6 (9.48) | |
| **Radiotherapy** | | | 0.87 [b] | | | 0.63 [b] |
| None | 139 (52 %) | 362 (52 %) | | 103 (54 %) | 297 (52 %) | |
| Radiotherapy | 131 (48 %) | 333 (49 %) | | 87 (46 %) | 272 (48 %) | |
| **Adjuvant therapy** | | | 0.024 [c] | | | 0.86 [c] |
| None | 238 (88 %) | 648 (93 %) | | 174 (92 %) | 523 (92 %) | |
| Endocrine therapy | 23 (9 %) | 39 (6 %) | | 14 (7 %) | 38 (7 %) | |
| Chemotherapy | 6 (2 %) | 4 (1 %) | | 1 (1 %) | 6 (1%) | |
| Both | 3 (1 %) | 4 (1 %) | | 1 (1 %) | 2 (0 %) | |
| **Tumor size**, mm | | | 0.051 [d] | | | 0.43 [d] |
| Median (Q1-Q3) | 12 (10-18) | 12 (10-15) | | 13 (9-17) | 12 (10-16) | |
| **TNM stage** | | | 0.058 [b] | | | 0.40 [c] |
| T1aN0M0 | 10 (4 %) | 19 (3 %) | | 2 (1 %) | 17 (3 %) | |
| T1bN0M0 | 85 (32 %) | 255 (37 %) | | 65 (34 %) | 190 (34 %) | |
| T1cN0M0 | 140 (52 %) | 366 (53 %) | | 102 (54 %) | 307 (54 %) | |
| T2N0M0 | 33 (12 %) | 51 (7 %) | | 21 (11 %) | 50 (9 %) | |
| Missing data | 2 | 4 | | 0 | 5 | |
| **Subtype** | | | <0.001 [b] | | | 0.88 [b] |
| Luminal A-like | 130 (49 %) | 416 (61 %) | | 109 (58 %) | 310 (55 %) | |
| Luminal B-like | 77 (29 %) | 179 (26 %) | | 51 (27 %) | 164 (29 %) | |
| HER2 positive | 17 (6 %) | 45 (7 %) | | 12 (6 %) | 42 (7 %) | |
| Triple negative | 40 (15 %) | 38 (6 %) | | 15 (8 %) | 48 (9 %) | |
| Missing data | 6 | 17 | | 3 | 5 | |
| **Histologic grade** | | | <0.001 [b] | | | 0.70 [b] |
| Grade I | 25 (10 %) | 117 (17 %) | | 28 (15 %) | 76 (14 %) | |
| Grade II | 134 (52 %) | 426 (63 %) | | 118 (62 %) | 336 (60 %) | |
| Grade III | 101 (39 %) | 133 (20 %) | | 44 (23 %) | 146 (26 %) | |
| Missing data | 20 | 19 | | 0 | 11 | |

[a] independent sample t-test, [b] chi square test, [c] Fisher's exact test, [d] Wilcoxon rank-sum test

SD – standard deviation, Q1-Q3 – interquartile range

Table S3. Univariable competing-risks regression depending on nuclear WRAP53 (C1-, C2-antibody) and RNA levels for IBTR within 10 years and BCD within 15 years.

| | **WRAP53 protein (C1)** | | **WRAP53 protein (C2)** | | ***WRAP53* RNA** | |
|---|---|---|---|---|---|---|
| | SHR (95 % CI) | p-value | SHR (95 % CI) | p-value | SHR (95 % CI) | p-value |
| **IBTR** | | | | | | |
| **WRAP53** | | | | | | |
| High (ref) | 1.00 | | 1.00 | | 1.00 | |
| Low | 1.87 (1.33-2.63) | **<0.001** | 1.30 (0.91-1.84) | 0.15 | 1.27 (0.86-1.90) | 0.23 |
| **Radiotherapy** | | | | | | |
| No radiotherapy | 1.00 | | 1.00 | | 1.00 | |
| Radiotherapy | 0.36 (0.25-0.52) | **<0.001** | 0.36 (0.25-0.52) | **<0.001** | 0.36 (0.25-0.52) | **<0.001** |
| **BCD** | | | | | | |
| **WRAP53** | | | | | | |
| High (ref) | 1.00 | | 1.00 | | 1.00 | |
| Low | 2.17 (1.52-3.10) | **<0.001** | 1.31 (0.90-1.91) | 0.16 | 1.17 (0.77-1.79) | 0.46 |

IBTR – ipsilateral breast tumor recurrence, BCD – breast cancer death, SHR – subhazard ratios, CI – confidence interval, ref – reference.

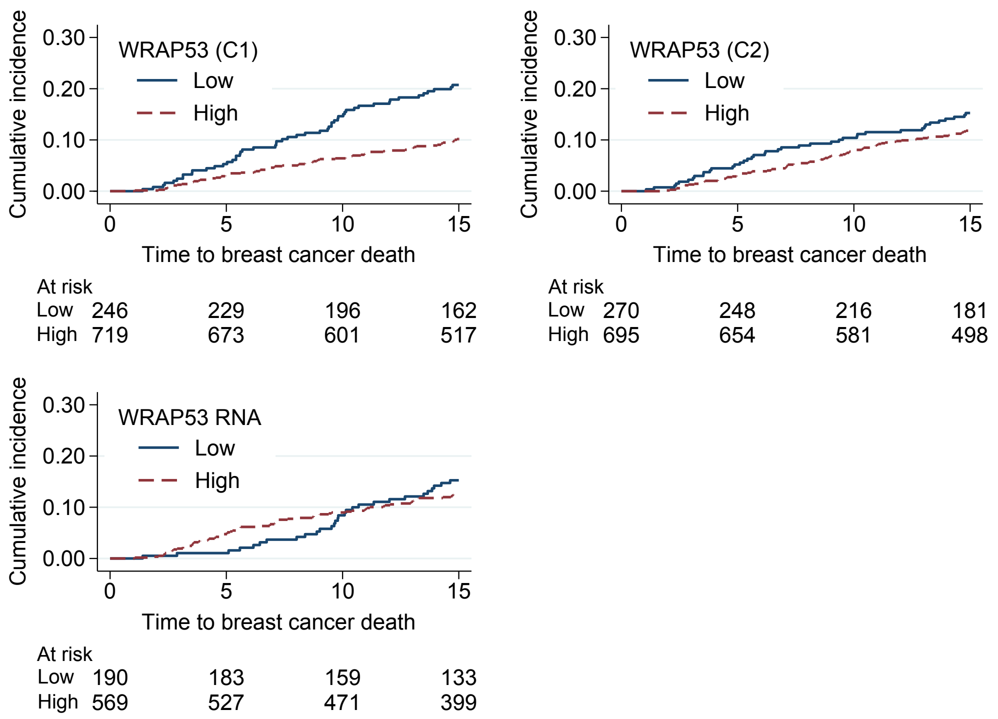


Figure S3. Cumulative incidence functions of time to breast cancer death in 15 years depending on WRAP53 levels (C1- and C2-antibody) and *WRAP53* RNA expression.

Table S4. Absolute events in IBTR- and BCD-competing risks analysis stratified by WRAP53 levels (C1- and C2-antibody) presented as total number (% of all). Competing risks in IBTR-analysis were any other recurrence and death. Competing risk in BCD-analysis was death from other cause than breast cancer.

| | **WRAP53 protein (C1)** | | **WRAP53 protein (C2)** | | ***WRAP53* RNA** | |
|---|---|---|---|---|---|---|
| | Low | High | Low | High | Low | High |
| **Events** | | | | | | |
| IBTR within 10 years | 54 (22 %) | 90 (13 %) | 47 (17 %) | 97 (14 %) | 34 (18 %) | 80 (14 %) |
| Competing events | 43 (17 %) | 110 (15 %) | 51 (19 %) | 102 (15 %) | 24 (13 %) | 89 (16 %) |
| Alive | 149 (61 %) | 519 (72 %) | 172 (64 %) | 496 (71 %) | 132 (69 %) | 400 (70 %) |
| | | | | | | |
| BCD within 15 years | 51 (21 %) | 73 (10 %) | 41 (15 %) | 83 (12 %) | 29 (15 %) | 73 (13 %) |
| Competing events | 33 (13 %) | 127 (18 %) | 46 (17 %) | 114 (16 %) | 28 (15 %) | 95 (17 %) |
| Alive | 162 (66 %) | 519 (72 %) | 183 (68 %) | 498 (72 %) | 133 (70 %) | 401 (70 %) |

IBTR – ipsilateral breast tumor recurrence, BCD – breast cancer death.

Table S5. Multivariable competing-risks regression depending on three levels of nuclear WRAP53 (C1- and C2- antibody) for IBTR within 10 years and BCD within 15 years. Tumors are divided depending on histoscores into low (histoscores 0-6), medium (8), and high (9-12). For C1 the distribution was: Low 246 tumors (25 %), Intermediate 443 (46 %), and High 276 (29 %). For C2 it was Low 270 (28 %), Intermediate 444 (46 %), and High 251 (26 %). In IBTR-analysis also adjusting for adjuvant therapy, subtype, histologic grade, tumor size and age. In BCD-analysis also adjusting for subtype, histologic grade, and tumor size.

| | **WRAP53 protein (C1)** | | **WRAP53 protein (C2)** | |
|---|---|---|---|---|
| | SHR (95 % CI) | p-value | SHR (95 % CI) | p-value |
| **IBTR** | | | | |
| **WRAP53** | | | | |
| Low, ref | 1 | | 1 | |
| Intermediate | 0.52 (0.31-0.87) | **0.013** | 0.70 (0.42-1.17) | 0.18 |
| High | 0.66 (0.38-1.15) | 0.14 | 1.13 (0.66-1.96) | 0.65 |
| **RT** | | | | |
| No RT, ref | 1 | | 1 | |
| RT | 0.44 (0.23-0.83) | **0.011** | 0.52 (0.27-0.99) | **0.046** |
| **Interaction** | | | | |
| Intermediate – RT | 0.92 (0.38-2.25) | 0.86 | 0.70 (0.28-1.74) | 0.44 |
| High - RT | 0.84 (0.32-2.20) | 0.72 | 0.61 (0.23-1.59) | 0.31 |
| **BCD** | | | | |
| **WRAP53** | | | | |
| Low, ref | 1 | | 1 | |
| Intermediate | 0.68 (0.43-1.06) | 0.086 | 1.13 (0.75-1.71) | 0.55 |
| High | 0.58 (0.33-1.01) | 0.056 | 0.67 (0.38-1.17) | 0.16 |

RT - radiotherapy, IBTR – ipsilateral breast tumor recurrence, BCD – breast cancer death, SHR – subhazard ratios, CI – confidence interval, ref – reference.

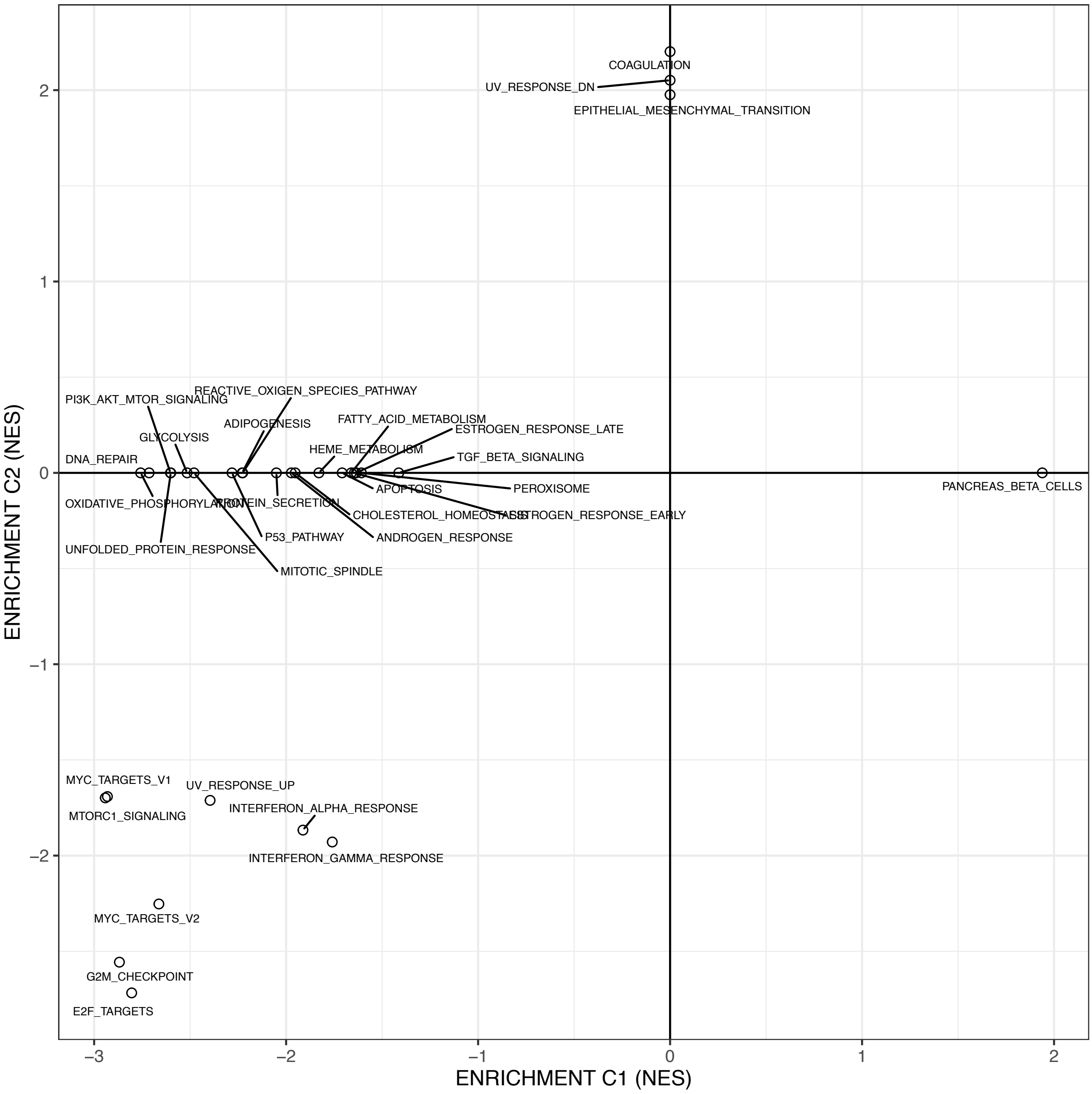


Figure S4. Scatter plot over gene pathways (Hallmarks database) enriched in tumors depending on WRAP53 protein levels (low and high) according to C1- and C2-antibody. Pathways enriched in both antibodies are seen in the bottom left section of the plot. NES – Normalized enrichment score.

Table S6. Top 20 genes significantly correlating with *WRAP53* RNA expression using Spearman's rank correlation.

| Gene names | Spearman's rho | p-value |
| --- | --- | --- |
| WRAP53 | 1,00 | <0,001 |
| ZFPM1 | 0,39 | <0,001 |
| AATK | 0,39 | <0,001 |
| MYO15A | 0,39 | <0,001 |
| HAGHL | 0,39 | <0,001 |
| CRB2 | 0,38 | <0,001 |
| NAPRT | 0,38 | <0,001 |
| GPR123 | 0,38 | <0,001 |
| MAST1 | 0,38 | <0,001 |
| SHISA8 | 0,38 | <0,001 |
| LIME1 | 0,38 | <0,001 |
| COMTD1 | 0,37 | <0,001 |
| PDZD7 | 0,37 | <0,001 |
| NOTUM | 0,37 | <0,001 |
| ABCA7 | 0,37 | <0,001 |
| CHTF18 | 0,37 | <0,001 |
| NKX24 | 0,37 | <0,001 |
| KNDC1 | 0,37 | <0,001 |
| FAM171A2 | 0,37 | <0,001 |
| RAD9A | 0,37 | <0,001 |
